# Supplementary material for: Utilising the Implementation of Integrated Care to Develop a Pragmatic Framework for the Sustained Uptake of Service Innovations (SUSI)
Source: Healthcare (Basel). 2023 Jun 16;11(12):1786. doi: 10.3390/healthcare11121786 (PMC10298662; doi:10.3390/healthcare11121786)
Supplement: Supplementary file 1 [file healthcare-11-01786-s001.zip › healthcare-2254883-supplementary.pdf]

Table S1. Summarised data. Evidence (literature, clinical guidelines) + clinical expertise (PAR study on the process of change from independent to IC in AOD&MH services).

**Research evidence (summarised).**	**Clinical and consumer expertise (summarised) [34].**		
	Participants	***Data collected***	***Key outcomes***
Phases of successful change [30-33].<br>• Establish motivation.<br>- Unfreeze, create urgency.<br>• Build support for change (& a change team).<br>• Manage the change transition.<br>- Develop a vision and strategies.<br>- Remove obstacles and create goals.<br>• Sustain Momentum.<br>- Refreeze. Make the change stick.<br><br>*Components of successful change [30-33].*<br>• Structured change management.<br>• Active executive sponsorship.<br>• Visible change leaders.<br>• Agreed scope & accountabilities.<br>• Resource availability.<br>• Clear and regular communication.<br>• Regular stakeholder engagement.<br><br>*IC implementation elements [16-22].*<br>• Work within capacity.<br>• No wrong door principal.<br>• Recognised as core business.<br>• Regular consumer engagement.<br>• Feedback from peers, carers, families.<br>• Collaborative service relationships.<br>• Flexibility and responsiveness to cultural and individual differences.<br>• Realistic expectations, holistic approach<br>• Client-centred, trauma-informed.<br>• Shared professional development.<br>• Monitor and evaluate.	i) PAR members, comprising 53 clinicians and managers from two AOD and MH services, and 91 residents of the non-acute residential MH unit.<br><br>ii) Clinical research team, comprising 4 self-nominated clinicians based in participating services (50% AOD).<br><br>iii) Research advisory group, comprising 6 invited reps. with research expertise, 1 AOD&MH Director, & the 4 members of the clinical research team.	• Barriers and solutions to implementing IC (focus groups, meeting minutes, survey data).<br><br>• Core phases and key enablers of the change process (focus groups and observations).<br><br>• Levels of contact and support between services, confidence to deliver IC, (surveys and observations).<br><br>• Levels of staff enthusiasm, productivity, and collaboration (meeting minutes and observational ratings).	• Momentum was sustained throughout the 34-month change process.<br><br>• Perceived barriers to IC included lack of understanding about the partnering service, varying levels of proficiency and confidence between services, and recurring workplace changes such as staff secondments and turnover of visiting medical specialists.<br><br>• **Key change enablers included visible permission from the top down (executive managers) and active participation from the frontline up (clinicians and service managers)/ presenting a challenge to be solved rather than a solution developed elsewhere/ allowing sufficient time to build genuine professional relationships between staff/ fostering meaningful contribution to clinical procedures/ and creating opportunities for staff to devise their own activities and methods.**<br><br>• **Solutions included regular meetings and training across services, communication between services from initial client engagement, e.g., pairing of workers for peer-mentoring and collaborative care-planning.**<br><br>• **Four critical phases for implementation of IC were identified: i) establishing active participation from managers and clinicians, ii) developing purposeful relationships between staff, developing ownership, and a shared vision, iii) co-designing a shared model; and iv) developing sustainability mechanisms at treatment and workforce levels and confidence to deliver new procedures.**<br><br>• The level of collaboration between staff increased through the process, as did levels of confidence. There were greater increases for AOD staff due to a smaller workforce and a higher percentage of staff taking part.

NOTE: the bold blue text indicates the key findings from the PAR study that are relevant to the development of the SUSI framework.
